# Dynamic Evolution‐Controlled Parabolic‐Shaped Microstructures for Ultra‐Black Surface via Self‐Assembled Microsphere Mask Etching

**DOI:** 10.1002/advs.76029

**Published:** 2026-06-09

**Authors:** Yiming Li, Jianwei Wang, Guoxu Yu, Qunbo Lv, Yuan Ma, Jiadao Wang

**Affiliations:** ^1^ State Key Laboratory of Tribology in Advanced Equipment Tsinghua University Beijing P. R. China; ^2^ Department of Mechanical Engineering Tsinghua University Beijing P. R. China; ^3^ Aerospace Information Research Institute Chinese Academy of Sciences Beijing P. R. China; ^4^ School of Physics and Optoelectronic Engineering Beijing University of Technology 100 Pingleyuan, Chaoyang District Beijing China

**Keywords:** broadband antireflection, microsphere mask etching, sidewall curvature control, ultra‐black surfaces

## Abstract

Suppressing broadband stray light remains a persistent obstacle in advanced optical instrumentation, where conventional blackening treatments often fall short in spectral range, surface conformity, or substrate compatibility. Here we report a fabrication approach that directly forms parabolic‐shaped microstructures on a blackout‐ink coating through self‐assembled microsphere mask etching. The process is governed by a time‐dependent shadowing evolution around the microspheres, which enables continuous tuning of the sidewall curvature—a structural parameter that is difficult to access using conventional micro/nanofabrication and emerges as a key determinant of optical attenuation. The method requires only spray‐coating of the ink followed by dry etching and applies to the surfaces of glass, metal, polymer, and other engineering materials. Uniform microstructure formation is maintained across planar and curved surfaces without lithography or substrate‐dependent optimization. The parabolic‐shaped structures yield an average reflectance 0.89% over 300–1700 nm, enabled by continuous refractive‐index grading and efficient photon trapping associated with the controlled sidewall profile. This work establishes a practical route for producing broadband ultra‐black surfaces on real optical components while revealing a previously unrecognized mechanism linking microsphere‐mediated morphological evolution to macroscopic optical suppression. The approach offers a straightforward and broadly applicable pathway for improving stray‐light management in precision metrology, imaging, and spaceborne systems.

## Graphical abstract

A robust, substrate‐independent fabrication approach overcomes the spectral and geometric limitations of traditional stray‐light suppression. By combining ink spray‐coating with self‐assembled microsphere mask etching, this method produces parabolic‐shaped microstructures on diverse planar and curved engineering components. The resulting continuous refractive‐index gradient delivers an exceptional average reflectance of 0.89% across the 300–1700 nm range, providing a practical route for high‐performance optical instrumentation.

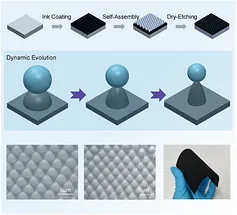

## 1 Introduction

In advanced optical systems—such as precision metrology [1, 2, 3], spaceborne remote sensing [4, 5], and quantum communication [6] — effective suppression of stray light is essential for maintaining detection sensitivity and distinguishing weak signals. Stray light arises from external background illumination as well as internal diffuse scattering and residual reflections, and its accumulation can obscure faint targets or saturate detectors [7]. Yet current surface‐treatment technologies remain insufficient for the ultra‐low‐reflectance requirements of modern instrumentation. Black anodizing, though robust and scalable for metallic components, still exhibits 8%–12% [8] reflectance at normal incidence and exceeds 20% at grazing angles. Commercial blackout inks used on glass surfaces offer convenient coating but typically yield ∼8% [9, 10] visible reflectance.

In weak‐signal applications, these reflection levels compound across multiple interfaces, limiting measurement precision. As a result, there is a pressing need for surface‐modification strategies that deliver broadband, angle‐stable, and substrate‐universal antireflective performance [11, 12, 13]. Ultra‐black surfaces, generally referring to surfaces exhibiting extremely low broadband reflectance at the percent level, have therefore emerged as a promising pathway for achieving advanced stray‐light suppression [14, 15, 16, 17].

In recent years, micro/nano structures have emerged as a key strategy for fabricating ultra‐black surfaces due to their ability to induce destructive optical interference, waveguide coupling, and diffuse scattering [18, 19, 20, 21]. For example, Qi et al. achieved over 99% absorption in the 400–700 nm range on metallic surfaces using femtosecond laser processing [22]. Zhong et al. fabricated nanostructures on silicon through chemical etching, achieving an average reflectance of 2.5% over 400–1100 nm [23]. Sun et al. employed inductively coupled plasma (ICP) etching to produce composite nanostructures on highly oriented pyrolytic graphite, resulting in reflectance below 1% across the 200–2000 nm range [24].

However, these methods remain tightly bound to specific substrates and geometries [22, 24, 25]. Their incompatibility with heterogeneous optical assemblies—composed of metals, glasses, and polymers—has limited the deployment of ultra‐black technologies in real engineering systems [26, 27]. While spray‐coating approaches can be applied to various substrates, coatings alone struggle to achieve the extremely low reflectance required for high‐performance optical systems [28, 29, 30, 31]. In addition, previously reported flexible ultra‐black surfaces generally involve trade‐offs among reflectance, scalability, fabrication complexity, mechanical robustness, and substrate versatility. A comparative analysis of representative flexible ultra‐black strategies is summarized in Table S1 [13, 24, 32, 33]. As a result, bridging ultra‐low reflectance, broadband coverage, and substrate universality remains an unresolved challenge at the materials–optics interface.

In this work, we introduce a fabrication approach that overcomes this longstanding constraint by forming micro/nanostructures directly on a widely used optical blackout‐ink coating. Instead of tailoring nanostructures for each substrate, we treat the ink layer itself as the etch medium, allowing identical processing conditions for glass, metal, polymer, and curved components.

Building on established principles of reflection suppression through nanostructuring [34, 35], we employ a self‐assembled microsphere mask etching (MME) strategy and show that the shadowing effect of the microspheres evolves dynamically during etching, giving rise to well‐defined parabolic microstructures. This time‐dependent evolution provides deterministic control over the sidewall curvature—an architectural parameter that is rarely accessible in conventional nanofabrication routes yet proves crucial for establishing continuous refractive‐index grading and enabling efficient photon trapping.

The proposed strategy breaks through the long‐standing limitation of “single‐substrate compatibility” inherent to conventional ultra‐black fabrication methods. It achieves consistent ultra‐black performance across heterogeneous substrates within complex optical assemblies, thereby filling a critical technological gap in substrate‐compatible ultra‐black engineering. Beyond the universality of the process, the dynamic evolution of microsphere shadowing during etching enables precise tuning of the sidewall curvature of the resulting microstructures—a morphological parameter that has been largely overlooked in previous studies. This capability allows us to directly correlate sidewall profiles with optical response, revealing the decisive role of curvature‐governed refractive‐index grading and photon‐trapping pathways in achieving extremely low reflectance. Moreover, the process is low‐cost and inherently scalable, significantly lowering the barrier for industrial deployment of ultra‐black coatings.

The fabricated composite ultra‐black surfaces exhibit an average reflectance of 0.89% across the ultraviolet‐to‐near‐infrared range, with excellent omnidirectional antireflective properties. This study provides a unified and easily implementable ultra‐black surface solution for high‐precision optical systems, while greatly expanding the substrate adaptability of ultra‐black materials. The approach holds strong potential to advance next‐generation technologies in weak‐signal detection, spaceborne remote sensing, and other advanced optical applications.

## 2 Results and Discussion

### 2.1 Validation of the Stamping Lithography process on a planar surface

After surface activation, a GT‐7 ink layer was spray‐coated onto the pretreated substrate and cured at 150°C for 1 h. This curing process strengthened the adhesion between the ink layer and the substrate while improving the mechanical robustness of the coating, thus enhancing its abrasion resistance under practical conditions.

**FIGURE 1:**
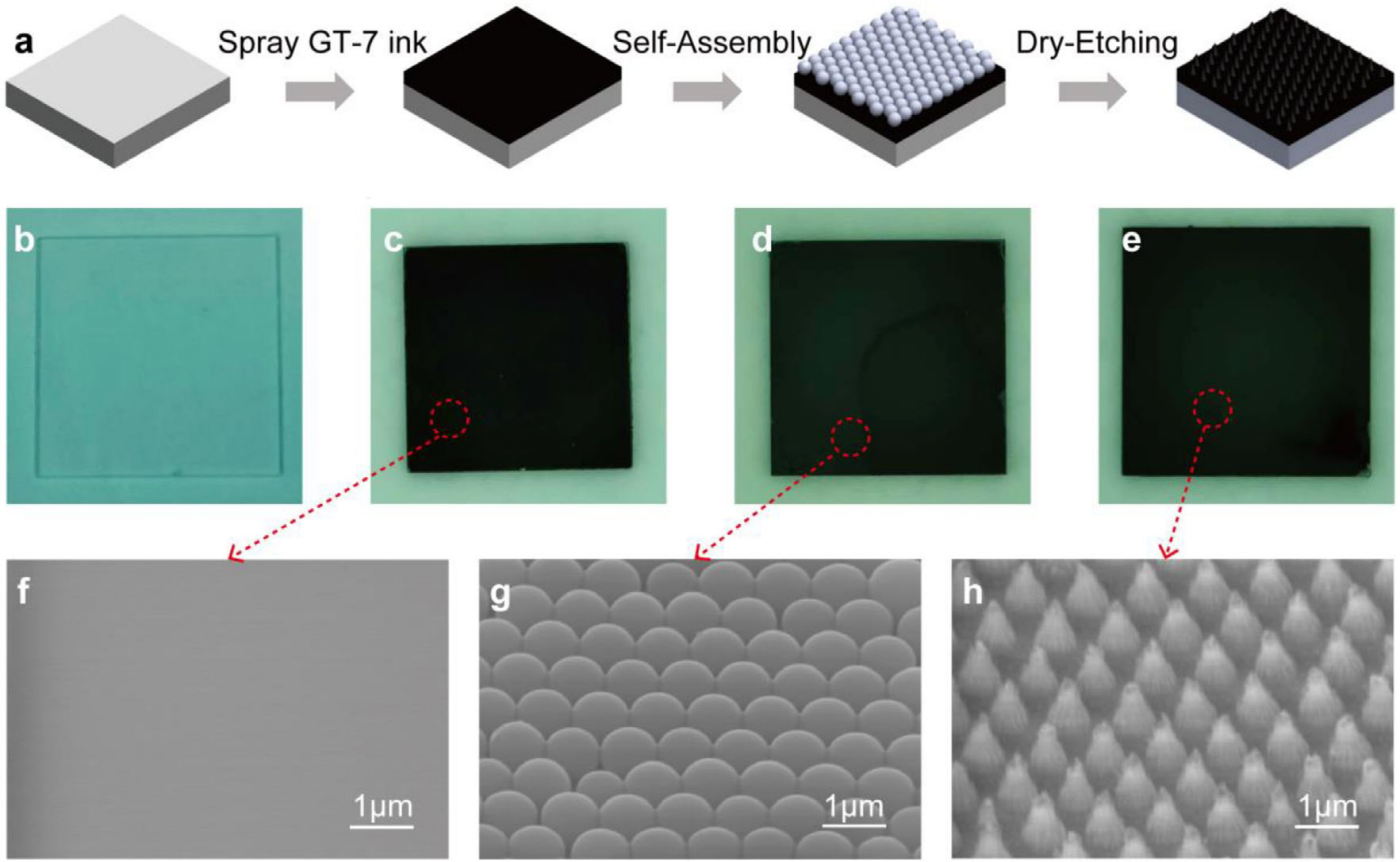
Fabrication and characterization of the ultra‐black surface. (a) Schematic illustration of the fabrication procedure of the ultra‐black surface. (b) Photograph of the glass substrate. (c) Image of the substrate after coating with GT‐7 ink. (d) Image of the surface after self‐assembly of SiO_2_ microspheres on the cured ink. (e) Image of the sample after dry etching. (f) SEM image showing the surface morphology of the cured ink. (g) SEM image showing the closely packed SiO_2_ microspheres obtained by self‐assembly. (h) SEM image displaying the nanocone array formed after dry etching.

The fabrication procedure of the ultra‐black surface is illustrated in Figure 1a. The substrates were first subjected to multistep ultrasonic cleaning in acetone, ethanol, and deionized water for 10 min each, effectively removing surface oils, micron‐scale particulates, and organic residues. Subsequently, plasma treatment (100 W, 30 s) was performed to enhance the surface hydrophilicity, thereby facilitating uniform spreading of the ink and improving interfacial adhesion in the following coating step.

A monolayer of silica microspheres was then assembled on the cured ink surface through a liquid–air interfacial self‐assembly process to serve as an etching mask. Inductively coupled plasma (ICP) etching was subsequently carried out using a mixed gas source of C_4_F_8_ and O_2_, optimized to match the etching selectivity between the epoxy‐based GT‐7 matrix and the silica microsphere mask. During plasma etching, C_4_F_8_ generates reactive fluorine species, which can react with SiO_2_ to form volatile SiF_4_, while simultaneously producing fluorocarbon species that contribute to surface passivation, resulting in a dynamic balance between etching and passivation [36]. Importantly, the selectivity between SiO_2_ and the substrate allows controllable mask evolution under different plasma conditions, enabling tunable morphology formation. This approach enabled precise morphology control guided by the microsphere template. After etching, the samples were ultrasonically cleaned to fully remove the residual microspheres (Figure S1).

Representative photographs of intermediate stages in the fabrication process are shown in Figure 1b–e, illustrating the evolution of the surface appearance at each step. Raman spectroscopy was employed to monitor the chemical composition during fabrication. As shown in Figure S2, the highly similar Raman profiles before and after etching verify that the intrinsic chemical structure remains preserved without introducing any impurities or phase transitions. The surface morphologies were characterized using scanning electron microscopy (SEM). The initial morphology of the cured ink is shown in Figure 1f. As shown in Figure 1g, the self‐assembled silica microspheres formed a closely packed, hexagonally ordered monolayer on the ink surface. During ICP etching, the measured etching rate was approximately 420–570 nm/min under the employed conditions. After ICP etching, a highly ordered array of nanocone structures was obtained (Figure 1h). The cone dimensions were comparable to those of the original silica microspheres, confirming accurate pattern transfer and controllable fabrication of the designed micro/nano structures. The detailed formation process of the microstructure is provided in Figure S3.

### 2.2 Anti‐Reflection Mechanism of Micro/Nano Structures

**FIGURE 2:**
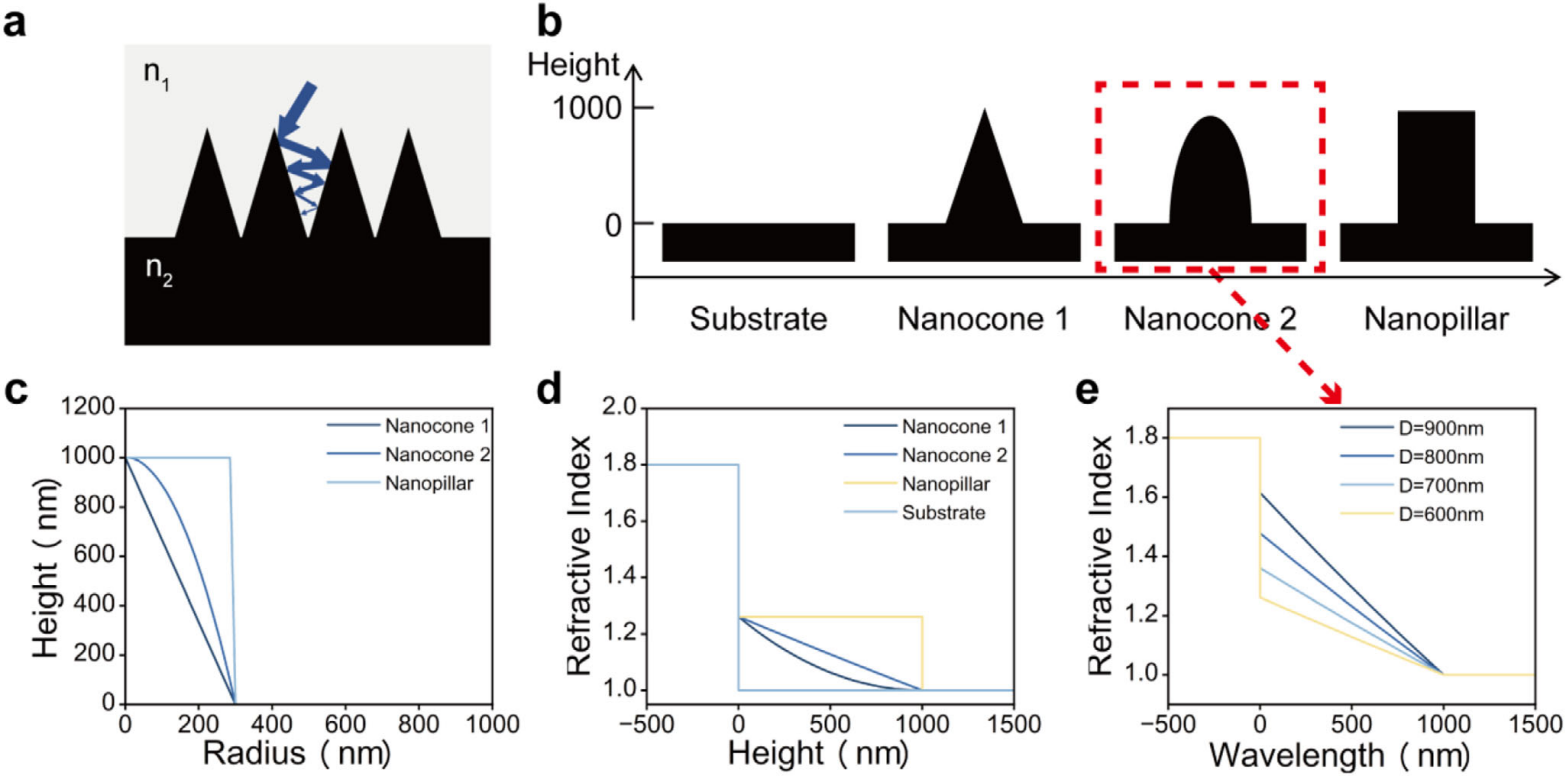
Effective refractive index analysis of different micro/nano structures. (a) Schematic of light incident on a micro/nanostructured surface. (b) Cross‐sectional schematics of the substrate and three representative structures. (c) Sidewall profile curves of the three structures. (d) Variation of the effective refractive index with height for the substrate and the three structures (Period: 900 nm) (e) Effective refractive index profiles of Nanocone 2 for different base diameters (Period: 900 nm).

The antireflection mechanism of micro/nano structures is generally understood as follows: when incident light impinges on the micro/nano structured surface, multiple reflections and scatterings occur within the interstitial gaps of the structures (Figure 2a). These interactions substantially enhance the absorption efficiency of the underlying material and ultimately lead to a pronounced reduction in reflectance [35, 37].

The reflectance at an interface is governed by the refractive indices of the two adjoining media. To describe how interfacial reflection depends on these indices, we use the Fresnel reflection equation (Equation 1) as the theoretical basis. When monochromatic light is normally incident from a homogeneous and isotropic medium with refractive index n_1_ onto a second medium with refractive index n_2_, and absorption or scattering at the interface is neglected, the reflectance can be approximated by:

(1) $$R={\left(\frac{n_{2}-n_{1}}{n_{2}+n_{1}}\right)}^{2}$$

Unlike a flat interface between two homogeneous media, a micro/nano structured surface presents a three‐dimensional topology. This transforms the interface into a complex optical system, making its reflectance far more difficult to quantify.

To elucidate the structure–property relationship between the morphology of the micro/nano structures and their antireflective performance, an analytical framework was established based on the effective medium theory (EMT). In this framework, the micro/nano structure array is treated as a medium with a graded refractive index [25, 38], and its key optical parameters are determined using an effective refractive index model. Specifically, the calculation of the effective refractive index employs the extended form of the Stavenga equation (Equation 2) [39]. Rooted in EMT, this formulation enables the periodic nanostructures to be accurately approximated as a homogeneous medium with a spatially varying refractive index.

(2) $$n_{eff}\;\left(h\right)={\left[f\left(h\right)\times n_{sub}^{q}+\left(1-f\left(h\right)\right)\times n_{air}^{q}\right]}^{1/q}\;$$

In (Equation 1), n_eff_ (h) denotes the effective refractive index at a structural height h; f(h) represents the filling factor, i.e., the area fraction of the structural material at that height; n_sub_ and n_air_ are the refractive indices of the substrate and air, respectively; and Q is a fitting parameter in the effective medium approximation. In this study, Q was set to 2/3, a value that has been validated by previous experimental and theoretical investigations, ensuring the reliability of this approximation.

Based on the effective refractive index model, the relationship between micro/nano structural morphology and its antireflective performance was analyzed. Three representative micro/nano structure models‐namely, nanocone(nanocone 1), nanocone with parabolic sidewall (nanocone 2), and nanopillar—were selected, with a planar surface serving as the reference group. The schematic cross‐sections of these models are illustrated in Figure 2b.

Figure 2c further depicts the geometric relationship between the core structural parameters of the three structures, showing the variation of sidewall height as a function of base radius.

Using the effective medium theory (EMT) and (Equation 2), the effective refractive indices of the four configurations (three structures and the planar surface) were calculated, as shown in Figure 2d. The results reveal the distribution of the effective refractive index as a function of structural height. Compared with the nanopillar and the nanocone 1, the nanocone 2 (with parabolic sidewalls) exhibits a more gradual refractive index gradient along the height direction. A smoother refractive index transition effectively suppresses interfacial index discontinuities during light incidence, thereby reducing reflection losses. Consequently, the nanocone 2 morphology demonstrates superior antireflective characteristics.

Furthermore, based on the nanocone 2 geometry, the influence of base diameter on the effective refractive index was investigated while maintaining a constant period and height. The calculated results, obtained from (Equation 2), are presented in Figure 2e. As the base diameter increases, the refractive index gradient becomes smoother, and the discontinuity decreases, indicating a more continuous transition. According to (Equation 1), a more gradual variation in the effective refractive index corresponds to lower reflectivity. In the following section, a detailed numerical analysis is performed to further explore the influence of geometric parameters on the optical response of the micro/nano structures.

### 2.3 Effect of Structural Parameters on Anti‐Reflective Performance

**FIGURE 3:**
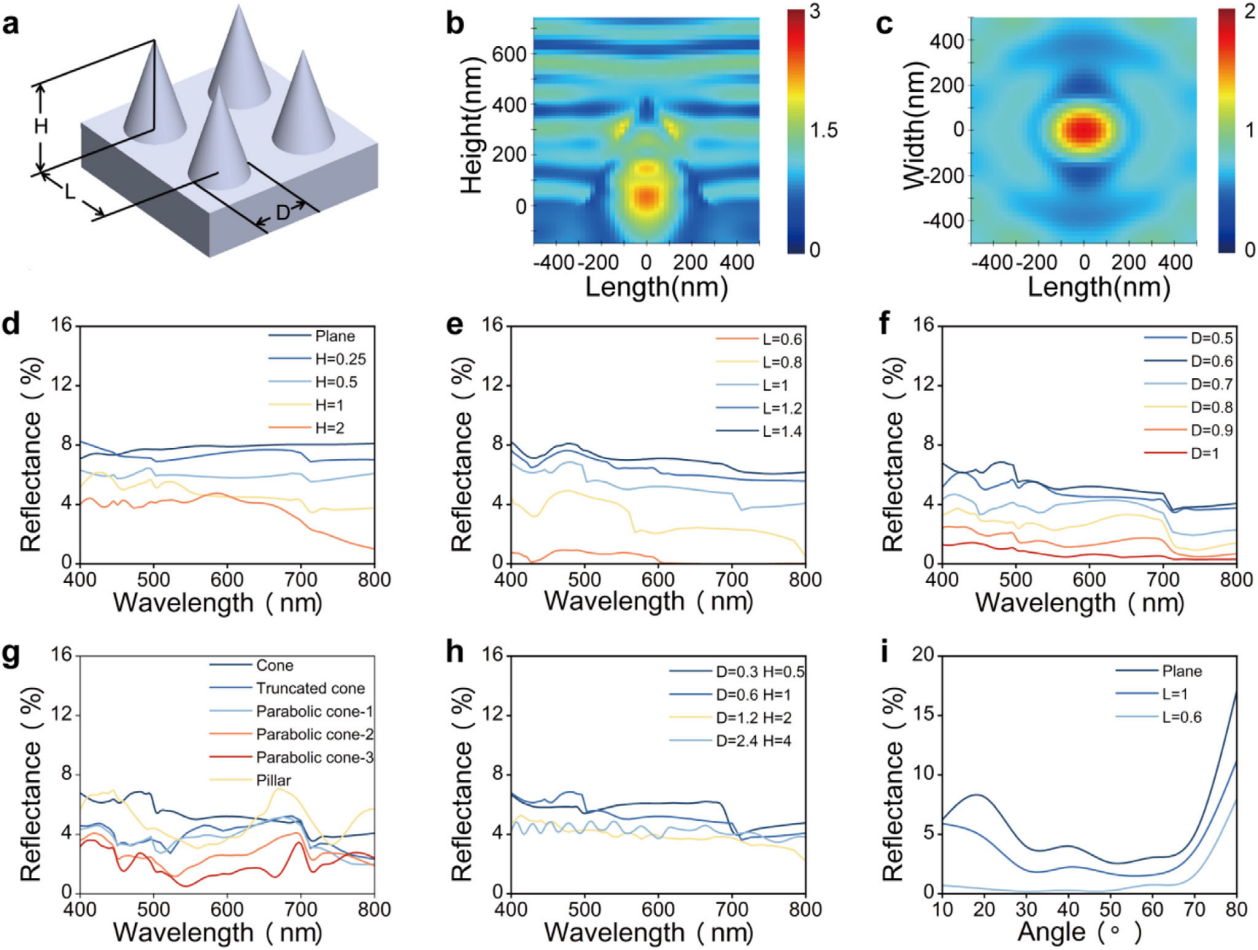
Numerical simulations of anti‐reflective micro–nanostructures. (a) Schematic illustration of the simulation model and geometric parameters. (b) Cross‐sectional and (c) top‐view distributions of the simulated electric field within the nanocone structure. (d–h) Simulated effective refractive index profiles under single‐variable conditions: (d) height,(e) period, (f) bottom diameter, (g) morphology, and (h) feature size. (i) Simulated reflectance spectra at different incident angles.

The antireflective performance of micro/nano structures exhibits a pronounced dependence on their geometrical parameters, including feature height, base diameter, periodicity, cross‐sectional morphology, and aspect ratio. To quantitatively elucidate this structure–property relationship and its underlying physical mechanisms, a numerical simulation model was established based on the finite‐difference time‐domain (FDTD) method, following the principles of optical field regulation. The representative micro/nano structure model used for simulation is illustrated in Figure 3a.

To evaluate the independent influence of each geometric parameter, a controlled‐variable approach was adopted to analyze five key factors: structure height, base diameter, periodicity, cross‐sectional profile, and characteristic aspect ratio.

As shown in Figure 3d, increasing the structure height significantly enhances the antireflective performance. Physically, taller structures form a smoother effective refractive index gradient between air and substrate, thereby reducing reflection losses caused by abrupt refractive index discontinuities.

Figure 3e illustrates the influence of periodicity. A smaller periodic arrangement markedly reduces surface reflectance because dense packing strengthens light scattering and coupling, increases the effective filling factor, and smooths the refractive index transition across the air–substrate interface, collectively contributing to reduced reflection.

The effect of base diameter is presented in Figure 3f. With increasing diameter, the antireflective efficiency improves, attributed to enhanced continuity of the filling factor, which facilitates a more gradual refractive index transition and further suppresses interfacial reflection.

The influence of cross‐sectional morphology on the antireflective performance is shown in Figure 3g. Six representative structures were comparatively analyzed, with their schematic models provided in Figure S4. The results indicate that the parabolic geometry exhibits the most superior antireflective capability. This observation is in excellent agreement with theoretical predictions derived from the Stavenga model, confirming the accuracy and reliability of the numerical simulation.

The effect of characteristic size, defined as the ratio of top to base diameter, is shown in Figure 3h. When the characteristic size is comparable to the incident wavelength, larger values enhance light coupling and absorption within the structure, thereby improving the antireflective effect.

Moreover, the proposed micro/nano structures exhibit outstanding omnidirectional antireflection—a crucial feature for practical optical systems. As shown in Figure 3i, the reflectance of structures with 1 µm and 600 nm periods remains consistently lower than that of the flat substrate within an incidence angle range of 0°–80°, validating their angular stability and effectiveness for real‐world applications.

### 2.4 Optimization of Dry Etching Parameters and Performance Tuning

Based on the structure–property relationship between micro/nano structure geometry and antireflective performance established through the aforementioned numerical simulations, an etching‐time‐dependent experiment was first conducted to verify the feasibility of morphology control using large‐sized microsphere masks. In this experiment, silica (SiO_2_) microspheres with a diameter of 4 µm were employed as the etching mask in a dry etching process. Compared with smaller microspheres, the 4 µm particles require a longer etching duration, thereby providing a broader temporal control window for precisely tuning key geometrical parameters such as structure height and base diameter. This makes them particularly suitable as an experimental model for investigating the correlation between etching time, morphology evolution, and optical performance.

**FIGURE 4:**
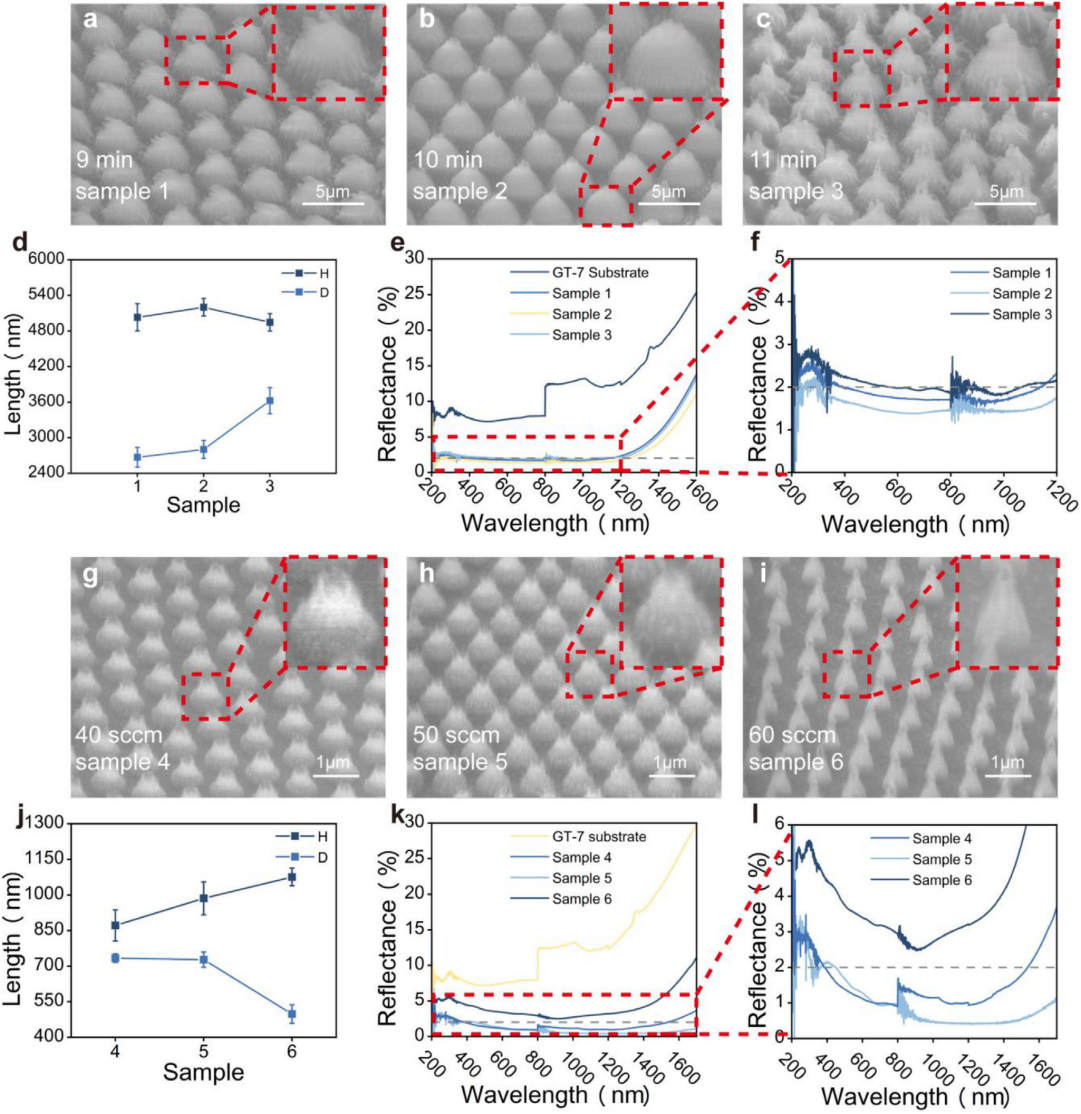
SEM characterization and reflectance measurements of microsphere‐masked etched surfaces. (a–c) SEM images of surfaces etched using 4 µm SiO_2_ microsphere masks; etch time varied as (a) 9, (b) 10, and (c) 11 min. Dashed boxes indicate regions shown at higher magnification to highlight morphology evolution with etch time. (d) Statistical distributions of geometric parameters (height and base diameter) for samples 1–3. (e, f) Total hemispherical reflectance spectra of samples 1–3. (g–i) SEM images of surfaces etched using 900 nm SiO_2_ microsphere masks; O_2_ flow rate varied as (g) 40, (h) 50, and (i) 60 sccm. (j) Statistical distributions of geometric parameters for samples 4–6. (k, l) Total hemispherical reflectance spectra of samples 4–6. All SEM images were acquired with the sample stage tilted by 45°. Fluctuations observed near ∼200 and ∼800 nm result from spectrometer source switching and are not intrinsic to the samples.

In the experiment, all processing parameters—such as etching power and gas flow ratios—were kept constant, except for the etching duration, which was set to 9, 10, and 11 min, respectively. The corresponding scanning electron microscopy (SEM) images of the micro/nano structures are shown in Figure 4a–c, and the statistical distributions of their geometrical dimensions (including structure height and base diameter) are summarized in Figure 4d.

According to the simulation results discussed earlier—namely, that a smoother refractive index gradient corresponds to enhanced antireflective performance—the structures obtained with a 10‐min etching duration (sample 2) exhibit a more favorable match between height and base diameter. This morphology facilitates a gradual refractive index transition along the vertical direction, which is expected to yield superior antireflective performance. Reflectance measurements (Figure 4e,f) confirmed this hypothesis.

It is worth noting that the fluctuations observed near 200 nm and 800 nm originate from the instrumental error associated with switching light sources between the UV–vis and vis–NIR regions in the spectrometer, rather than from any intrinsic properties of the samples. After accounting for this systematic deviation, the reflectance of sample 2 remains below 2% throughout the 300–1200 nm spectral range, demonstrating a significantly better performance compared to the other etching conditions.

Although the 4 µm SiO_2_ microsphere mask experiment confirmed the tunable relationship between etching duration and the antireflective performance of the micro/nano structures, and achieved a relatively low reflectance, the micrometer‐scale feature size still exceeds the wavelength of visible light. Consequently, the optical modulation precision and performance ceiling across a broad spectral range—particularly in the near‐infrared region—remain constrained.

To overcome this limitation, SiO_2_ microspheres with a diameter of 900 nm were subsequently employed as the etching mask. The selection of this particle size was guided by the following considerations: since the 900 nm scale is comparable to the wavelength range of interest (visible to near‐infrared), the resulting structures can significantly enhance light scattering according to Mie scattering theory. Moreover, by precisely tuning the arrangement and geometrical parameters of these submicron structures, more efficient reflectance suppression can be achieved, providing a theoretical foundation for surpassing the performance limitations of micrometer‐scale morphologies.

In the 900 nm SiO_2_ microsphere mask system, the effect of a key process parameter—gas flow rate—was further investigated. On one hand, the optimal etching duration had already been determined in the preceding 4 µm microsphere experiments; on the other hand, the gas flow rate, which strongly influences plasma activity, plays a more critical role in the precise formation of nanoscale features, particularly in controlling height uniformity and top‐surface morphology.

Therefore, establishing a quantitative correlation among gas flow rate, structural dimensions, and optical performance is essential for refining the controllable fabrication of micro/nano structures. In this study, the C_4_F_8_ flow rate was fixed at 20 sccm, while the O_2_ flow rate was varied to 40, 50, and 60 sccm. The corresponding SEM images are shown in Figure 4g–i, and the statistical results of structural dimensions are summarized in Figure 4j.

The results reveal that as the O_2_ flow rate increases, the height of the micro‐nanostructures increases significantly, while the base diameter gradually decreases. This trend can be attributed to the enhanced plasma reactivity: with higher O_2_ flow, the concentration of reactive oxygen species (O^+^ and O_2_^+^) increases, accelerating the etching rates of both the microsphere mask and the substrate material. Consequently, the etching front progresses more rapidly in the vertical direction, resulting in taller structures and reduced lateral protection at the base.

Further examination of the SEM morphologies reveals that the structures fabricated under 40 sccm (sample 4) and 50 sccm (sample 5) exhibit relatively smooth top surfaces, whereas those etched at 60 sccm (sample 6) display sharper apex features. According to the previously established simulation conclusion—that a smoother top surface facilitates a gradual refractive index transition and minimizes reflection losses—it can be inferred that sample 5 should exhibit the best antireflective performance.

Reflectance measurements (Figure 4k,l) confirmed this hypothesis. The reflectance of sample 5 remained extremely low reflectivity across the 300–1700 nm spectral range. Compared with the unstructured ink‐coated substrate, the average reflectance in the visible region (400–760 nm) was reduced from approximately 8% to below 1%, and that in the near‐infrared region (760–1700 nm) dropped from 10%–25% to below 1%. To verify fabrication reproducibility, three independent optical measurements were performed on the representative optimal sample (Sample 5). As shown in Figure S5, the highly overlapping reflectance spectra across 200–1700 nm confirm the excellent structural stability and data reliability of the prepared surfaces.

These results not only validate the optimization achieved using the 900 nm microsphere mask but also stand in sharp contrast to the outcomes of the 4 µm system, underscoring the superior broadband antireflective capability of nanoscale structures. Figure S6 shows that distributed etching can also produce nanopillar structures, highlighting the versatility of this method in controlling surface morphology. Collectively, this work demonstrates a clear progression from fundamental morphological control to a substantial performance breakthrough.

### 2.5 Multisubstrate Validation and Performance Evaluation

**FIGURE 5:**
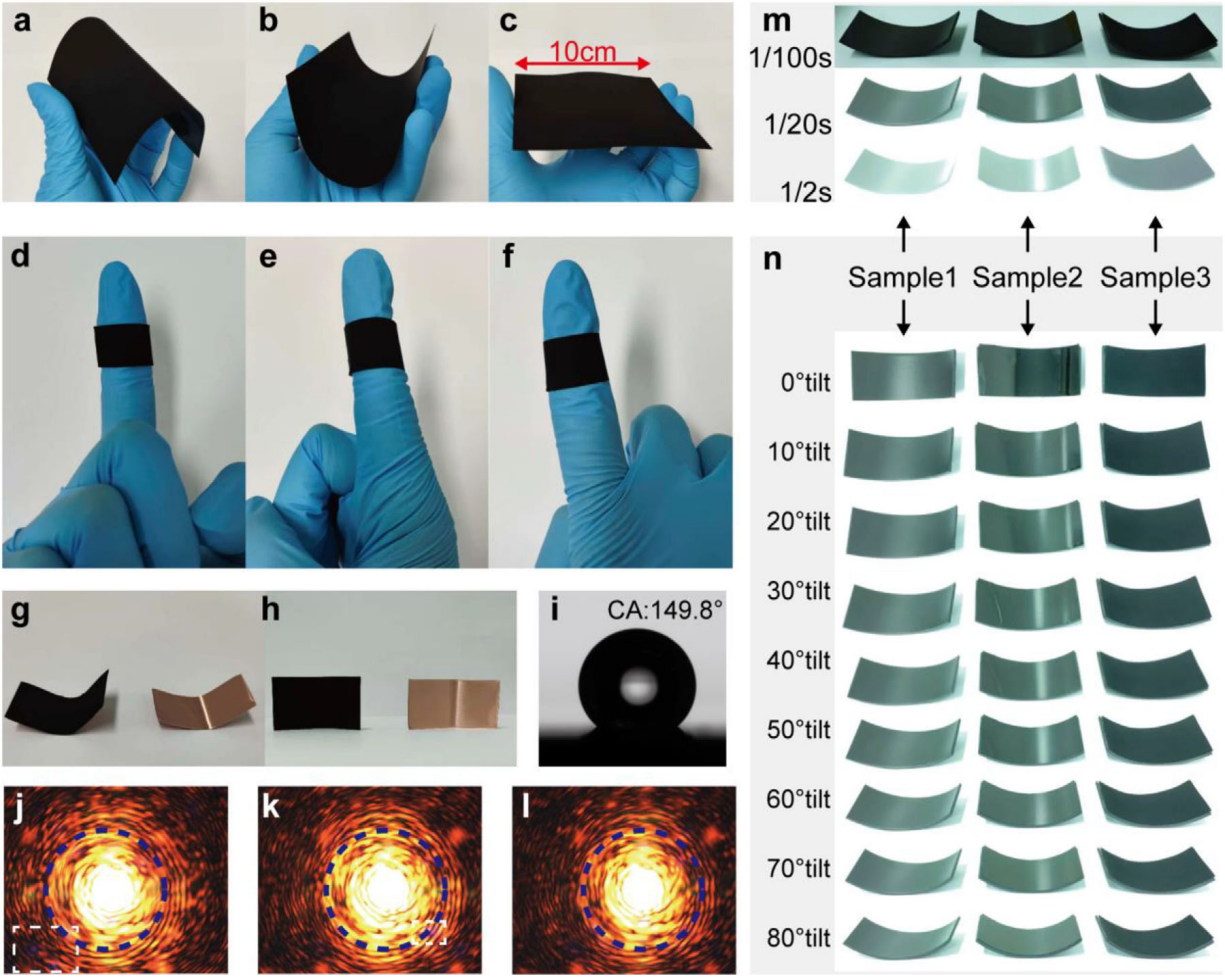
Universality and application performance of the ultra‐black surface. (a–c) Ultra‐black PI films under different mechanical states: bent outward (a), bent inward (b), and laid flat (c). (d–f) Multi‐angle views of the ultra‐black film conformally attached to a human finger, demonstrating mechanical flexibility. (g, h) Visual comparison between ultra‐black treated and pristine copper sheets. (i) Water contact angle measurement of the ultra‐black surface. (j–l) Laser‐spot images recorded by the same camera under three internal‐baffle conditions: untreated (j), blackout‐ink coated (k), and fully ultra‐black treated (l). (m) Images of the three baffle conditions at a 60° incident angle under different exposure times, highlighting differences in surface reflectance. (n) Images captured at a fixed exposure time of 1/20s over incident angles ranging from 0° to 80°. (sample 1: untreated; sample 2: blackout‐ink coated; sample 3: ultra‐black treated).

To test the universally applicability of this ultra‐black fabrication method, we applied it to different substrate materials and surface shapes. Figure 5a–c shows the results on flexible polyimide (PI) films. In both flat and bent states, the treated PI films display a uniform, deep‐black appearance with very low reflectance. When the films are attached to a finger, as shown in Figure 5d–f, they still maintain the same ultra‐black effect, indicating that the optical performance remains stable under complex bending.

We also treated the surface of a curved copper sheet. The results are shown in Figure 5g,h. After processing, the bright metallic gloss is fully suppressed. From the viewing angle in Figure 5h, the folded region of the copper sheet can hardly be distinguished, suggesting that the treatment effectively hides the surface shape. These results confirm that the method is compatible with metal substrates and provides strong anti‐reflection performance across different materials.

It is also worth noting that the etched micro/nano structures give the surface strong hydrophobicity. The contact‐angle measurement in Figure 5i shows that six repeated tests at different positions (Figure S7) yield an average contact angle of 145.68°, with a maximum of 149.8°, approaching the threshold for superhydrophobic behavior. This hydrophobicity provides the ultra‐black surface with effective anti‐fouling and self‐cleaning capabilities, further extending its potential use in harsh or contaminated environments. The mechanical durability and thermal tolerance of the fabricated surface were further evaluated. As shown in Figure S8, the coating exhibits excellent stability, maintaining Grade 0 adhesion in the cross‐cut tape test and preserving its original sub‐micron morphology after multiple thermal shock cycles up to 200°C.

The ultra‐black surface was further applied to the inner wall of a camera baffle (treatment region and procedure shown in Figure S9), and its ability to suppress stray light was evaluated using a laser spot test. Figure 5j–l shows images captured by the same camera under identical conditions (632 nm laser illumination and fixed imaging geometry). The three cases correspond to the untreated baffle (Figure 5j), the baffle coated only with blackout ink (Figure 5k), and the baffle after the full ultra‐black treatment (Figure 5l).

A clear improvement is observed across the three states. In the untreated configuration (Figure 5j), strong stray light appears outside the blue circular region. After the ultra‐black treatment (Figure 5l), the intensity outside this region becomes markedly weaker, indicating a significant enhancement in spot concentration. In addition, the white rectangular region in Figure 5j–k show pronounced chromatic stray‐light artifacts, whereas these artifacts disappear entirely in Figure 5l. This confirms that the ultra‐black surface effectively suppresses stray light and improves overall optical concentration.

Figure 5m,n compares the light‐absorption performance of lens hood in three states: untreated (left), coated only with blackout ink (middle), and processed with the full ultra‐black treatment (right). Figure 5m shows images obtained at a 60° incident angle under varying exposure times, whereas Figure 5n presents results acquired at 1/20 s exposure across incident angles from 0° to 80°. Additional data collected at 1/100 s and 1/2 s are provided in Figure S10.

Across all conditions, the ultra‐black–treated baffle exhibits a pronounced reduction in reflections. It consistently absorbs light reaching the inner wall and maintains this suppression over a broad range of illumination angles.

## 3 Conclusion

In response to the critical need for suppressing stray light and minimizing interface reflection in optical systems, we developed a universal fabrication strategy for ultra‐black surfaces based on a synergistic combination of MME and a blackout ink coating. This approach simultaneously achieves broadband anti‐reflection, multi‐substrate compatibility, and simplified fabrication. The resulting surfaces exhibit an average reflectance 0.89% across the ultraviolet‐to‐near‐infrared range, with a minimum reflectance of 0.4% in this region. Moreover, the process demonstrates exceptional substrate universality, being directly applicable to metals, glasses, and polymers without modification. The fabrication involves only two essential steps—spray coating of blackout ink and MME—without the need for complex lithography or vacuum processing, thus offering a cost‐effective and scalable route to large‐area ultra‐black surfaces.

Mechanistically, the anti‐reflective behavior of our surface follows the same physical principles reported in previous studies, where micro/nanostructural modulation serves as the core mechanism for reflection suppression. However, our approach introduces a significant conceptual innovation in fabrication strategy. Whereas most existing works have focused on constructing nanostructures on silicon or silica substrates, we target the ink layer itself—a material widely employed in optical systems for stray‐light reduction—as the modification medium. Direct etching of the ink layer forms dense arrays of nanocone structures, which not only endow the surface with exceptionally low reflectance but also extend the effective spectral response into the UV–vis–NIR regime. In contrast to substrate‐specific approaches, the present method does not require parameter tuning for different materials or geometries and can be uniformly applied to both planar and curved surfaces. This substantially enhances the adaptability of ultra‐black coatings in complex optical assemblies.

Notably, this study provides the first clear evidence that the sidewall profile of nanocone structures exerts a critical influence on anti‐reflective performance. Previous research has primarily focused on macroscopic geometric parameters such as height, period, and base diameter, while overlooking the effects of microscopic features like the curvature of sidewalls. The unique capability of the MME process to precisely tailor nanostructure profiles enables direct investigation of this factor. Such control is challenging to achieve using conventional photolithography, laser ablation, or nanoimprinting techniques. The discovery highlights that the optical response of micro/nanostructures depends not only on dimensional scaling but also on fine morphological details, offering new insight into the design principles of high‐efficiency anti‐reflective surfaces.

Although the average reflectance achieved in this work (0.89%) does not yet surpass the absolute record values reported for state‐of‐the‐art ultra‐black materials, it represents a well‐balanced optimization among four essential attributes—extremely low reflectance, broadband coverage, substrate universality, and cost‐effectiveness—which are typically mutually constrained. The ability to harmonize these factors within a single, scalable fabrication framework constitutes a distinct advantage over existing ultra‐black technologies.

In summary, we have established a “blackout ink–microsphere‐mask etching” hybrid strategy for the fabrication of universal ultra‐black surfaces. The process has been successfully applied to diverse substrates, including metals, glass, and polymers, as well as to both planar and curved geometries, confirming its broad applicability. The resulting surfaces achieve stable ultra‐low reflectance across the UV–vis–NIR spectrum through the synergistic action of graded refractive index transitions and efficient light trapping. This work bridges the gap between high optical performance and manufacturing versatility, providing a practical and scalable route for implementing ultra‐black coatings in advanced optical systems. The proposed approach offers new opportunities for the large‐scale deployment of ultra‐black materials in fields such as precision photonics, stray‐light suppression, and high‐sensitivity detection.

## 4 Experimental Section

### 4.1 The Preparation of the Ink Layer

The GT‐7 blackout ink (Canon Inc., Tokyo, Japan) consisted of a main agent, a hardener, and a diluent. The three components were thoroughly mixed by vigorous shaking for approximately 5 min prior to use. The ink was prepared at a weight ratio of 8:1:8 (main agent:hardener:diluent). Specifically, the hardener was added first and weighed, followed by the main agent. After stirring for 1 min, the diluent was introduced, and the mixture was further stirred for 10 min to ensure homogeneity.

The substrates were sequentially ultrasonicated in ethanol and deionized water for 5 min each, followed by plasma surface treatment (PT500, Jiangsu lebo science Inc.) to enhance hydrophilicity.

The prepared ink was spray‐coated onto the pretreated substrate using a commercially available hand‐held spray bottle, with a typical spray distance of ∼10–20 cm to ensure uniform coverage, followed by curing in a thermostatic oven at 150°C for 1 h. This curing condition was selected according to the manufacturer's recommendation, providing sufficient crosslinking while avoiding incomplete curing at lower temperatures or thermal degradation at higher temperatures. The curing process strengthened the adhesion between the ink layer and the substrate while improving the mechanical robustness of the coating, thus enhancing its abrasion resistance under practical conditions.

### 4.2 Assembly of SiO_2_ Microsphere Monolayers

The ink‐coated substrate was placed in a container filled with deionized water. A 2.5 vol% suspension of SiO_2_ microspheres (Tianjin Baseline Chromtech Research Centre) was introduced into the container via a syringe pump until the spreading of microspheres was no longer effective. Hydrogels containing sodium dodecyl sulfate (SDS) were placed in the water to assist microsphere assembly. After the formation of a close‐packed microsphere layer, the film was transferred onto the substrate by gradually lifting the substrate or lowering the water level.

### 4.3 Inductively Coupled Plasma Etching

All samples were fabricated using an inductively coupled plasma (ICP) etching system (NE‐550H, ULVAC, Japan). The standard etching conditions were as follows: RF power of 200 W, ICP power of 500 W, O_2_ flow rate of 20 sccm, and C_4_F_8_ flow rate of 40 sccm. For the SiO_2_ microspheres with a diameter of 900 nm, the typical etching duration was 60 s. The effects of etching parameters on the resulting structures were systematically investigated, and detailed conditions are provided in the previous section. After the ICP etching, all samples were ultrasonically cleaned in ethanol to remove surface residues.

### 4.4 Optical and Morphological Characterization

Spectrally resolved total reflectance was measured using a double‐beam UV–vis–NIR spectrophotometer equipped with an integrating sphere (Cary 5000, Agilent Technologies). The instrument was calibrated prior to each measurement to ensure data accuracy, and all reflectance spectra are presented as raw data.

Surface morphologies were characterized using a field‐emission scanning electron microscope (FEI QUANTA 200 FEG) operated at an acceleration voltage of 10 kV. Before imaging, the samples were coated with a ∼5 nm layer of Pt using a DC magnetron sputter coater with rotary–planetary tilting stages (EM ACE600, Leica Microsystems).

## Author Contributions

**Jiadao Wang**: data curation, supervision, project administration, writing – review and editing. **Yiming Li**: conceptualization, methodology, validation, writing – original draft. **Guoxu Yu**: methodology, visualization. **Qunbo Lv**: writing – review and editing, data curation, supervision. **Jianwei Wang**: methodology, validation, visualization, investigation. **Yuan Ma**: writing – review and editing, supervision, data curation, project administration.

## Funding

This work was supported by the National Key R&D Program·of China, 2022YFB3903000(2022YFB3903001) and the National Natural Science Foundation of China (NSFC) (Grant No. 52275200 and No. 52205312).

## Conflicts of Interest

The authors declare no conflicts of interest.

## Supporting information




**Supporting File 1**: advs76029‐sup‐0001‐SuppMat.docx.

**Supporting File 2**: advs76029‐sup‐0002‐Data.zip.

## Data Availability

The data that support the findings of this study are available from the corresponding author upon reasonable request.
