# Supplementary material for: Dynamic Evolution‐Controlled Parabolic‐Shaped Microstructures for Ultra‐Black Surface via Self‐Assembled Microsphere Mask Etching
Source: Adv Sci (Weinh). 2026 Jun 9;13(48):e76029. doi: 10.1002/advs.76029 (PMC13336935; doi:10.1002/advs.76029)
Supplement: Supplementary file 1 [file ADVS-13-e76029-s002.docx]

Supporting Information

Dynamic Evolution-Controlled Parabolic-shaped Microstructures for Ultra-Black Surface via Self-Assembled Microsphere Mask Etching

Yiming Li, Jianwei Wang, Guoxu Yu, Qunbo Lv*, Yuan Ma*, Jiadao Wang*

Yiming Li,Guoxu Yu,Yuan Ma, Jiadao Wang

Department of Mechanical Engineering, Tsinghua University, Beijing 100084, P.R. China

*Yuan Ma - Email: [yuanma@tsinghua.edu.cn](mailto:yuanma@tsinghua.edu.cn)

*Jiadao Wang - Email: [jdwang@mail.tsinghua.edu.cn](mailto:jdwang@mail.tsinghua.edu.cn)

*Qunbo Lv - Email:lvqb@aircas.ac.cn

Jianwei Wang, Qunbo Lv

Aerospace Information Research Institute Chinese Academy of Sciences, Beijing 100094, P.R. China

Yiming Li and Jianwei Wang contributed equally to this work and share first authorship.

**Supporting Table**

| **Method** | **Avg. Reflectance** | **Mechanical Stability** | **Cost** | **Substrate Versatility** | **Scalability** | **Manufacturing Efficiency** |
| --- | --- | --- | --- | --- | --- | --- |
| Graphite etched structures^[24]^ | 0.5% | Poor | High | Low | Limited | Low |
| CNT^[32]^ | 1.5% | Moderate | Very High | Low | Moderate | Low |
| PDMS with Nigrosine^[13]^ | 0.4% | Excellent | Low | Moderate | Moderate | Moderate |
| Carbon black/  Polyurethane-silica resin^[33]^ | 1.4% | Good | Low | High | Excellent | High |
| **This work (Ink spray + etching)** | **1.%** | **Excellent** | **Moderate** | **High** | **Excellent** | **High** |

**Table S1.Comparison of flexible ultra-black surfaces using different fabrication strategies.**

The reported average reflectance values were collected from the corresponding literature under different measurement conditions and spectral ranges; therefore, direct quantitative comparison should be interpreted cautiously.The qualitative evaluations of mechanical stability, substrate versatility, scalability, and manufacturing efficiency were comparatively assessed based on fabrication complexity, material compatibility, large-area processability, and structural robustness reported in the literature.

Although several previously reported methods exhibit lower reflectance, they generally suffer from limitations including high fabrication cost, poor scalability, or limited substrate compatibility. In contrast, the present strategy provides a balanced combination of low reflectance, mechanical robustness, substrate versatility, and scalable fabrication.

**Supporting Figures**

**Figure S1. SEM images of micro/nano structures after dry etching.** a,b) SEM images showing residual microspheres when the etching time is insufficient. c) Top-view SEM image revealing the periodic ordering of the etched micro/nanostructures.

When the dry etching time is insufficient to fully remove the microsphere mask, SEM imaging reveals residual microspheres on the top surfaces and within the gaps of the micro/nano structures. Typical examples are highlighted by the red circles in Figure S1a-b. In addition, the top-view SEM image (Figure S1c) shows a well-ordered periodic arrangement of the structures, confirming the uniform structural control achieved by the etching process.

**Figure S2. Raman spectra of the samples before and after etching.**

To further verify whether the etching process altered the chemical composition of the sample, Raman spectroscopy measurements were conducted before and after etching. The results show that the main characteristic Raman peaks remain nearly unchanged after etching, without noticeable peak disappearance, newly generated peaks, or significant peak shifts. This suggests that the etching process mainly modified the surface morphology while preserving the intrinsic chemical composition and phase structure of the material. These Raman results further confirm that no substantial chemical degradation or compositional transformation occurred during etching.

**Figure S3.Morphological evolution and SEM characterization during the formation of micro/nanostructures.** a) Schematic illustration of the etching-induced evolution, showing the full transition from the initial microsphere mask to the final nanocone-like geometry. b–e) SEM images acquired at different etching stages, providing direct experimental evidence for the morphological evolution outlined in a).

The formation of micro/nano structures using microspheres as an etching mask relies on the dynamic co-evolution between the mask and the substrate. During dry etching, the microspheres are progressively consumed, leading to a continuous reduction in their diameter. As the mask shrinks, the protected area on the underlying substrate contracts accordingly. This coupled “mask consumption–protection shrinkage” process drives the gradual emergence of nanocone-like features on the substrate surface, with the morphology evolving systematically as the etching time increases.

**Figure S4. Simulation model configuration.**

**Figure S5.Evaluation of Data Reproducibility.**

To evaluate the reproducibility of the fabrication process and the reliability of the optical measurements, three independent measurements were conducted on the representative optimal sample (Sample 5). As depicted in Figure S5, the three reflective spectra exhibit an exceptionally high degree of overlap across the entire wavelength range from 200 nm to 1700 nm. This minor fluctuation demonstrates the excellent structural stability of the prepared samples and the robust reliability of our experimental characterization system.

**Figure S6.** **Fabrication and optical characterization of pillar-like micro/nano structures.** a) Schematic illustration of the stepwise etching process, showing the two-stage evolution from microsphere resizing to substrate etching. b–e) SEM images of pillar structures fabricated under different etching parameters (varying microsphere size and total etching time). f–g) Statistical distributions of structural dimensions. h–i) Reflectance measurements of the resulting pillar arrays.

Pillar-like micro/nano structures were fabricated using a stepwise etching strategy enabled by the etching selectivity of different reactive gases. In the first stage, tetrafluoromethane (C₄F₈) was employed as the etchant. Owing to its significantly higher etch rate for SiO₂ compared with the matt-ink substrate, C₄F₈ allows the SiO₂ microspheres to be precisely downsized while imparting minimal etching to the underlying substrate. Once the microsphere size reaches the target value, the process transitions to the second stage, in which oxygen (O₂) is introduced as the etchant. O₂ exhibits strong etching activity toward the blackout ink substrate but only weakly etches SiO₂. The reduced microspheres thus function as protective masks for the substrate directly beneath them, while the exposed regions between adjacent microspheres are gradually removed by O₂, yielding well-ordered pillar-like features on the substrate surface.

Based on the reflectance measurements and structural characterization, the variation in reflectance among the samples is primarily governed by the geometry of the microstructures. Sample 4 exhibits the lowest average reflectance, which corresponds to its relatively high aspect ratio (~5:1) compared with the other samples (approximately 1:1–2:1). A higher aspect ratio enhances multiple light scattering and increases the effective optical path length, leading to improved light trapping and reduced reflectance.Sample 3 shows reduced inter-structure spacing, resulting in a larger effective bottom coverage within the same structural period. This configuration also contributes to lower reflectance by increasing the probability of light interaction with the microstructured surface. These observations are consistent with the simulation results, which indicate that the reflectance strongly depends on both aspect ratio and spatial filling density of the microstructures.

This stepwise approach reliably produces anti-reflective pillar structures that are distinctly different from the cone-like features obtained through a single-step etch. The method offers an expanded tuning window, enabling independent control of the SiO₂ mask size and the pillar height/diameter by decoupling the two etching stages. It also supports enhanced structural diversity, providing greater flexibility for designing micro/nano structures tailored to specific functional requirements. Owing to its reliance on etching selectivity, the approach is broadly extendable to other “mask–substrate” material combinations, offering a versatile platform for the scalable fabrication and multifunctional integration of micro/nano structures.

**Figure S7. Contact-angle measurements of the ink layer before and after the ultra-black treatment.**

**Figure S8. Evaluation of the mechanical and thermal stability of the sample.** a, b) Optical photographs of the sample surface a) before and b) after the cross-cut tape test conducted according to the ISO standard, demonstrating Grade 0 adhesion. c) Infrared thermal image of the sample during the thermal shock test at an elevated temperature of 200 ℃. d) Top-view SEM image of the surface microstructure after 5 consecutive thermal shock cycles between room temperature and 200 ℃, showing no obvious morphological degradation.

To evaluate the mechanical durability and practical reliability of the prepared coating, a standard cross-cut tape test was implemented in accordance with the ISO 2409 standard. As illustrated in Figures S8a and S8b, no detachment or peeling of the coating flakes was observed along the edges or within the grid squares after the taping process, classifying the adhesion strength at the highest rank of Grade 0. This outstanding adhesion performance is intrinsically attributed to the ink-based nature of the precursor, which undergoes cross-linking and robust chemical/physical bonding with the underlying substrate during the high-temperature curing process.

Furthermore, a rigorous thermal shock resistance test was executed to assess the structural integrity of the coating under extreme temperature fluctuations. The sample was rapidly heated from room temperature to 200°C (Figure S8c), sustained at this elevated temperature for 5 min, and subsequently cooled back to room temperature. After enduring 5 consecutive thermal cycles, the macro-appearance of the sample remained intact without any cracking or delamination. More importantly, the scanning electron microscopy (SEM) image (Figure S8d) verifies that the sub-micron conical arrays on the surface preserved their original ordered morphology without any thermally-induced collapse or distortion, confirming the exceptional thermal stability of the fabricated microstructure.

**Figure S9. Ultra-black treatment of the camera baffle and spot-pattern testing.** a) Photograph of the ultra-thin PI film after the ultra-black treatment (initial PI thickness: 50 μm). b) Camera image prior to the ultra-black modification. c) Camera image after applying the ultra-black-treated PI film. d) Optical setup used for spot-pattern measurement.

To accommodate the curved inner surface of the camera baffle and ensure ease of processing, an ultrathin polyimide (PI) film (50 μm in thickness) was selected as the substrate. The PI film was first subjected to the ultra-black treatment described in the main text, and the processed film was subsequently laminated onto the inner wall of the camera baffle. Spot-pattern measurements were performed using the optical setup shown in Figure S9d. Three baffle conditions were evaluated—untreated, PI film coated with blackout ink, and PI film after the ultra-black treatment—to compare their respective abilities to suppress stray light.

**Figure S10.Multi-angle imaging of lens hoods at different exposure times.** a) Images of the three lens hood states acquired at various tilt angles under an exposure time of 1/100 s. b) Corresponding images recorded at an exposure time of 1/2 s.

The three lens hood configurations—Sample 1: untreated, Sample 2: PI film laminated after ink coating, and Sample 3: PI film laminated after the ultra-black treatment—were imaged at varying exposure times while the hoods were tilted to different angles. The resulting image brightness provides a direct indicator of the hood’s antireflection performance: darker appearances correspond to stronger absorption of incident light and thus improved suppression of reflections.
